# Ventricular assist devices as bridge to heart transplantation: impact on post-transplant infections

**DOI:** 10.1186/s12879-016-1658-0

**Published:** 2016-07-08

**Authors:** Delphine Héquet, Georg Kralidis, Thierry Carrel, Alexia Cusini, Christian Garzoni, Roger Hullin, Pascal R. Meylan, Paul Mohacsi, Nicolas J. Mueller, Frank Ruschitzka, Piergiorgio Tozzi, Christian van Delden, Maja Weisser, Markus J. Wilhelm, Manuel Pascual, Oriol Manuel, Swiss Transplant Cohort Study (STCS), Rita Achermann, Patrizia Amico, John-David Aubert, Philippe Baumann, Guido Beldi, Christian Benden, Christoph Berger, Isabelle Binet, Pierre-Yves Bochud, Elsa Boely, Heiner Bucher, Leo Bühler, Thierry Carell, Emmanuelle Catana, Yves Chalandon, Sabina de Geest, Olivier de Rougemont, Michael Dickenmann, Michel Duchosal, Laure Elkrief, Thomas Fehr, Sylvie Ferrari-Lacraz, Christian Garzoni, Paola Gasche Soccal, Christophe Gaudet, Emiliano Giostra, Déla Golshayan, Karine Hadaya, Jörg Halter, Dominik Heim, Christoph Hess, Sven Hillinger, Hans H. Hirsch, Günther Hofbauer, Uyen Huynh-Do, Franz Immer, Richard Klaghofer, Michael Koller, Bettina Laesser, Roger Lehmann, Christian Lovis, Oriol Manuel, Hans-Peter Marti, Pierre Yves Martin, Luca Martinolli, Pascal Meylan, Paul Mohacsi, Philippe Morel, Ulrike Mueller, Nicolas J Mueller, Helen Mueller-McKenna, Antonia Müller, Thomas Müller, Beat Müllhaupt, David Nadal, Manuel Pascual, Jakob Passweg, Chantal Piot Ziegler, Juliane Rick, Eddy Roosnek, Anne Rosselet, Silvia Rothlin, Frank Ruschitzka, Urs Schanz, Stefan Schaub, Aurelia Schnyder, Christian Seiler, Susanne Stampf, Jürg Steiger, Guido Stirnimann, Christian Toso, Christian Van Delden, Jean-Pierre Venetz, Jean Villard, Madeleine Wick, Markus Wilhelm, Patrick Yerly

**Affiliations:** Transplantation Center, University Hospital (CHUV) and University of Lausanne, Lausanne, Switzerland; Infectious Diseases Service, University Hospital (CHUV) and University of Lausanne, Lausanne, Switzerland; Institute for Clinical Epidemiology and Biostatistics, University Hospital of Basel, Basel, Switzerland; Clinic for Cardiovascular Surgery, Inselspital, Bern University Hospital and University of Bern, Bern, Switzerland; Department of Infectious Diseases, Inselspital, Bern University Hospital and University of Bern, Bern, Switzerland; Clinic of Internal Medicine and Infectious Diseases, Clinica Luganese, Lugano, Switzerland; Department of Medicine, Service of Cardiology, University Hospital (CHUV) and University of Lausanne, Lausanne, Switzerland; Institute of Microbiology, University Hospital (CHUV) and University of Lausanne, Lausanne, Switzerland; Bern University Hospital and University of Bern, Bern, Switzerland; Division of Infectious Diseases and Hospital Epidemiology, University Hospital, University of Zurich, Zurich, Switzerland; Department of Cardiology, Cardiovascular Center, University Hospital, University of Zurich, Zurich, Switzerland; Department of Cardiovascular Surgery, University Hospital (CHUV) and University of Lausanne, Lausanne, Switzerland; Transplant Infectious Diseases Unit, University Hospitals Geneva, Geneva, Switzerland; Division of Infectious Diseases and Hospital Epidemiology, University Hospital Basel, Basel, Switzerland; Clinic for Cardiovascular Surgery, University Hospital Zurich, Zurich, Switzerland; Infectious Diseases Service and Transplantation Center, University Hospital and University of Lausanne, BH 10/553, CHUV, Lausanne, Switzerland

**Keywords:** Outcome, Cardiac transplantation, Mechanical heart support

## Abstract

**Background:**

Ventricular assist devices (VAD) are valuable options for patients with heart failure awaiting cardiac transplantation. We assessed the impact of pre-transplant VAD implantation on the incidence of post-transplant infections in a nationwide cohort of heart transplant recipients.

**Methods:**

Heart transplant recipients included in the Swiss Transplant Cohort Study between May 2008 and December 2012 were analyzed. Cumulative incidence curves were used to calculate the incidence of bacterial or *Candida* infections (primary endpoint) and of other infections (secondary endpoint) after transplant. Cox regression models treating death as a competing risk were used to identify risk factors for the development of infection after transplant.

**Results:**

Overall, 119 patients were included in the study, 35 with a VAD and 84 without VAD. Cumulative incidences of post-transplant bacterial or *Candida* infections were 37.7 % in VAD patients and 40.4 % in non-VAD patients. In multivariate analysis, the use of cotrimoxazole prophylaxis was the only variable associated with bacterial/*Candida* infections after transplant (HR 0.29 [95 % CI 0.15-0.57], p < 0.001), but presence of a VAD was not (HR 0.94, [95 % CI 0.38-2.32], p = 0.89, for continuous-flow devices, and HR 0.45 [0.15 – 1.34], *p* = 0.15, for other devices). Risk for post-transplant viral and all fungal infections was not increased in patients with VAD. One-year survival was 82.9 % (29/35) in the VAD group and 82.1 % (69/84) in the non-VAD group. All 6 patients in the VAD group that died after transplant had a history of pre-transplant VAD infection.

**Conclusion:**

In this nationwide cohort of heart transplant recipients, the presence of VAD at the time of transplant had no influence on the development of post-transplant infections.

**Electronic supplementary material:**

The online version of this article (doi:10.1186/s12879-016-1658-0) contains supplementary material, which is available to authorized users.

## Background

Ventricular assist devices (VAD) are an established option for patients suffering from end-stage heart failure that may not survive until a suitable donor comes [1]. A major complication associated with VAD support is the high incidence of infections of the devices, ranging from 25-70 % [2–4], which can be life-threatening and/or may jeopardize subsequent transplantation [5]. Some series show that *Staphylococcus spp.* and *Candida spp.* are the most important microorganisms involved in VAD infections [6, 7]. Any part of the device can be involved in these infections, including the driveline, the cannulae, the pocket or the pump itself [8, 9].

Large registries of patients have shown that mortality after heart transplantation is not increased in patients with VAD support, particularly when continuous-flow left ventricular devices are used [10, 11]. However, data on the impact of VAD implantation on the incidence of post-transplant infections are conflicting, in particular when the device is infected at the time of transplantation. While some studies reported that pre-transplant VAD-related infections correlated with a higher incidence of bacteremia following transplantation and a decreased survival [12, 13], in another study the presence of VAD increased the risk for local, but not for disseminated infections, and VAD infections were not associated with inferior outcomes [14]. Additionally, despite some reports suggesting that VAD may generate a relative state of immunosuppression after implantation [15, 16], there is a lack of current data reporting on the incidence of opportunistic viral or fungal infection in heart transplant recipients with pre-transplant VAD.

The Swiss Transplant Cohort Study (STCS) is a comprehensive prospective nationwide cohort including the majority of solid-organ transplantations (SOT) performed in Switzerland. We conducted an observational study of all heart transplant recipients included in the STCS to evaluate the impact of the implantation of a VAD on the incidence of post-transplant infections.

## Methods

### Patient population and study design

The STCS is a multicenter nationwide cohort study including more than 95 % of all solid-organ transplant recipients in Switzerland from May 2008 onward [17]. Three adult and pediatric heart transplant programs participate in the STCS: Bern, Lausanne-Geneva, and Zurich; these centers also perform most of the VAD implantations in Switzerland. For the present study, we included consecutive patients transplanted from May 2008 to December 2012 with at least one follow-up and written informed consent for participation in the STCS.

Data on demographic parameters, transplant type, comorbidities, immunosuppressive treatment, antimicrobial drugs, rejection, infectious and non-infectious events are collected at enrollment, at six months and every 12 months on standardized data forms. We used the STCS database for available data regarding the occurrence of infections, demographic and clinical data, as well as long-term graft function and survival. A specific questionnaire was used by the investigators to collect detailed pre-transplant data that were not included in the STCS database. Specifically, data such as the type of VAD (continuous-flow vs. others), date of implantation and duration of VAD support, INTERMACS (Interagency Registry for Mechanically Assisted Circulatory Support) score [18], incidence and cause of any pre-transplant infections (and whether the infection was considered as being currently active or cured), antibiotics used and their duration, blood transfusions, laboratory values (creatinine) and body mass index (BMI) were studied. All cases of death occurring after transplantation were reviewed by a clinician in charge of patients and a STCS investigator.

### Immunosuppressive and antimicrobial prophylaxis after transplantation

Immunosuppressive regimens varied among centers, but in general, all patients received, after an intraoperative methylprednisolone bolus, induction therapy (with either antithymocyte globulins [ATG] or basiliximab), followed by triple maintenance therapy with calcineurin inhibitors or mTOR inhibitors, an antimetabolite (mycophenolate or azathioprine) and steroids. Cotrimoxazole was administered for prevention of *Pneumocystis jiroveci* pneumonia and toxoplasmosis for a duration varying from 6 months to lifelong. Antifungal prophylaxis was not standardized and depended on specific individual risk factors. Prevention of cytomegalovirus (CMV) disease consisted in antiviral prophylaxis with valganciclovir for high-risk patients (donor positive/recipient negative for CMV). For CMV seropositive recipients, two programs administered antiviral prophylaxis and one program managed the patients by preemptive therapy [19].

### Clinical definitions

The definitions of infections in (heart) transplant recipients follow the guidelines of the Infectious Diseases Study Group of the STCS and are in concordance with the American Society of Transplantation recommendations for screening, monitoring and reporting of infectious complications in immunosuppression trials in recipients of organ transplantation [20]. Briefly, each episode of infection was reviewed by the local transplant infectious diseases specialist and classified in proven, probable, and possible infection according to standardized criteria. Asymptomatic episodes were classified as colonization (bacteria, fungus) or asymptomatic replication (virus) depending on the pathogen. Only proven/probable infections were included in the present analysis, except for CMV, for which all infections were included.

The definitions of infection in pre-transplant patients with VAD follow the guidelines from the International Society for Heart and Lung Transplantation [21]. VAD-specific infections are those related to the device hardware, which do not occur in non-VAD patients (e.g. pump, cannulae, percutaneous driveline, and pocket infections). VAD-related infections refer to infections that may also occur in patients who do not have VADs (e.g. infective endocarditis, bloodstream infections, mediastinitis). According to these definitions, an episode of infection may be defined as being both a VAD-specific and a VAD-related infection (e.g. in case of a bloodstream infection secondary to a pump pocket infection). Finally, non-VAD infections were defined as those not affected by the presence of the VAD, such as lower respiratory tract infection, cholecystitis, *Clostridium difficile* infection or urinary tract infection. Acute rejection was defined according to the original International Society for Heart and Lung Transplantation (ISHLT) Heart Biopsy Grading Scale [22].

### Statistical analysis

Descriptive analysis was used to determine the baseline and clinical characteristic of the subjects in the study. Univariate analysis was performed using partial tests of fitted univariable logistic regression models for categorical variables and continuous variables. Cumulative incidence curves were calculated by presence of VAD to estimate the probability of first bacterial or *Candida* infection after transplantation treating death prior to an infection as competing risk; Kaplan-Meier curves were calculated to estimate patient survival for patients with and without VAD. We chose, as a primary endpoint, only bacterial or *Candida* infection, because they are the most common infections developing after VAD implantation. We analyzed the incidence of other infections (viral, all fungal) as secondary endpoints.

Variables that might influence the development of infection after transplantation based on previous literature, such as implantation of VAD device, type of the VAD (continuous-flow vs. other), age, gender, prior diabetes, transplant center, donor age, donor sex, cotrimoxazole prophylaxis and type of immunosuppressive therapy were analyzed with a Cox proportional hazards regression model, taking into account death as a competing risk by using non-parametric multiple imputation techniques to recover the missing potential censoring information for individuals who died [23–25]. In the multivariable analyses, all covariates with missing information were imputed by the most frequent class (categorical covariates) or by the median of the available values (continuous covariates). A p-value of less than 0.05 was considered significant. All analyses were performed with R version 2.15.1 [26].

## Results

### Study population

Overall 119 patients (35 with a VAD and 84 without VAD) underwent heart transplantation between May 2008 and December 2012, and informed consent and at least one follow-up were available in all patients. Baseline characteristics are shown in Table 1. There were no significant differences between the two groups. Mean follow-up after transplantation was 1.36 years for the whole cohort. Immunosuppressive regimens were similar between groups.

**Table 1:** Baseline characteristics of heart transplant recipients included in the study

	No VAD (*n* = 84)	VAD (*n* = 35)
Follow-up, years; mean (SD)	1.38 (1.07)	1.31 (1.06)
Recipient age, years; mean (SD)	47.9 (16.9)	45.7 (16.7)
Recipient gender, male; n (%)	62 (73.8)	28 (80)
Donor age, years; mean (SD)	41.3 (16.5)	40.7 (15.0)
Donor gender, male; n (%)	48 (57.1)	24 (68.6)
Prior diabetes; n (%)	15 (17.9)	4 (11.4)
BMI, kg/m2; mean (SD)	24.4 (4.7)	25.2 (4.8)
Serum creatinine, umol/l; mean (SD)	125.9 (66.0)	103.3 (47.1)
Underlying disease; n (%)		
- Cardiomyopathy - Ischemic - Congenital heart disease - Other	41 (48.8) 23 (27.4) 5 (5.9) 15 (17.9)	18 (51.4) 12 (34.3) 1 (2.9) 4 (11.4)
Cold ischemia, min; mean (SD)	151 (54.3)	164 (54.4)
Bypass time, min; mean (SD)	168 (65.6)	186 (71.1)
Induction therapy; n (%)		
- Basiliximab - ATG - None	7 (8.3) 71 (84.5) 6 (7.1)	6 (17.1) 24 (68.6) 5 (14.3)
Maintenance at discharge; n (%)		
- Tacrolimus - Cyclosporin - MMF/MPA - Azathioprine - Everolimus - Steroids	28 (33.3) 39 (46.4) 63 (75.0) 21 (25.0) 9 (10.7) 74 (88.1)	10 (28.6) 13 (37.1) 22 (62.9) 7 (20.0) 3 (8.6) 27 (77.1)

There were no significant differences between the two groups. *VAD* ventricular-assist device, *BMI* body mass index, *ATG* anti-thymocyte globulin, *MMF* mycophenolate mofetil, *MPA* mycophenolic acid

### Pre-transplant characteristics of patients with VAD

Table 2 shows the characteristics of patients with VAD at the time of implantation according to the presence or not of a pre-transplant infection. The majority of the patients received a Heartmate®-II (40 %) or a Berlin Heart® EXCOR (34.3 %). The median INTERMACS score at the time of implantation was 2 (IQR 1–4). Median time from VAD implantation to transplantation was 161 days (IQR 75–354).

**Table 2:** Pretransplant characteristics at the time of VAD implantation according to pre-transplant VAD infection

Characteristics	VAD infection *n* = 18	No VAD infection *n* = 17
Age, years; mean (SD)	52.3 (9.7)	39.1 (20)
Gender, male; n (%)	15 (83.3)	14 (82.4)
BMI, kg/m2; mean (SD)	28.4 (6.1)	22.9 (6)
Median time from VAD implantation to transplantation, days; median (IQR)	293.5 (121–593)	101 (37.5-206)
INTERMACS score; median (IQR)	1.5 (1–4)	2 (1–3)
Creatinine, μmol/l; mean (SD)	105.3 (58.6)	90.9 (35.6)
Type of VAD; n (%)		
- Heartmate® II - Berlin Heart® EXCOR - Heartware® - Thoratec® - Levitronics®	9 (37.1) 6 (34.3) 1 (14.3) 2 (11.4) 0 (0.0)	5 (29.4) 6 (35.3) 4 (23.5) 1 (5.8) 1 (5.8)
Active infection at the time of transplant	10/18 (55.5)	-

*VAD* ventricular-assist device, *BMI* body mass index, *INTERMACS* interagency registry for mechanically assisted circulatory support

**Fig. 1:**
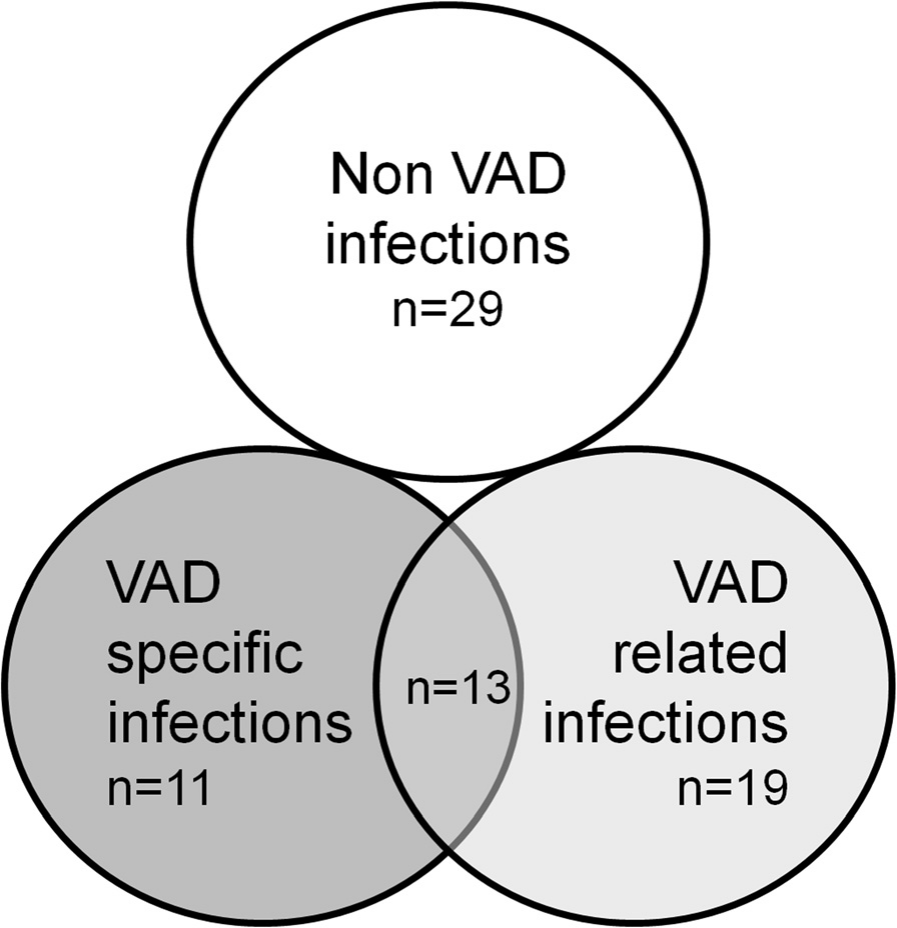
Number of episodes of pre transplant infection in patients with VAD according to the ISHLT classification

The incidence of infections from implantation to heart transplantation is shown in Table 3. Eighteen patients (51.4 %) developed 43 episodes of VAD-specific or related infections, with a median of one episode per patient (IQR 0–1). On note, three patients developed 11, five and four episodes of infection, respectively. Overall, 24/43 (55.8 %) episodes were classified as being VAD-specific infections and 32/43 (74.4 %) were VAD-related infections, so that 13/43 (30.2 %) were classified as being both VAD-specific and VAD-related infection (Fig. 1). Additionally, 29 episodes of non-VAD infection were reported (data not shown). Eight out of the 18 patients with VAD-specific or related infections were considered by the clinician in charge as being free from the VAD infection before transplantation, and they were not receiving antibiotic therapy at the time of transplantation (Additional file 1: Table S1).

**Table 3:** Type of pre-transplant infection in patient with a VAD

Infections	VAD patients *n* = 18
Total number of VAD-specific or related infections^a^	*n* = 43
VAD-specific infections; n (%) • *Proteus mirabilis* • *Enterobacter spp.* • *Staphylococcus aureus* • *MSSA* • *MRSA* • Coagulase-negative *Staphylococci* • *Escherichia coli* • *ESBL* • *Serratia marcescens* • *Enterococcus faecalis* • *VRE* • Other	24 (55.8) 6 (13.9) 4 (9.3) 3 (7.0) 2 (4.7) 1 (2.3) 3 (7.0) 3 (7.0) 0 (0) 2 (4.7) 2 (4.7) 0 (0) 8 (18.6)
VAD-related infections; n (%) • *Staphylococccus aureus* • *MSSA* • *MRSA* • Coagulase-negative *Staphylococci* • *Enterobacter spp.* • *Proteus mirabilis* • *Escherichia coli* • *ESBL* • *Enterococcus spp.* • *VRE* • *Candida spp.* • Other	32 (74.4) 7 (16.3) 2 (4.7) 5 (11.6) 3 (7.0) 7 (16.3) 5 (11.6) 3 (7.0) 0 (0) 3 (7.0) 0 (0) 1 (2.3) 4 (9.3)

*ESBL* extended spectrum betalactamase, *MSSA* methicillin susceptible *Staphylococcus aureus*, *MRSA* methicillin resistant *Staphylococcus aureus*, *VAD* ventricular-assist device, *VRE* vancomycin-resistant *Enterococci*

^a^In 13 cases, infection was classified as being both VAD-specific and VAD-related, therefore the addition of numbers is >100 %

### Impact of VAD implantation on post-transplant outcomes

**Fig. 2:**
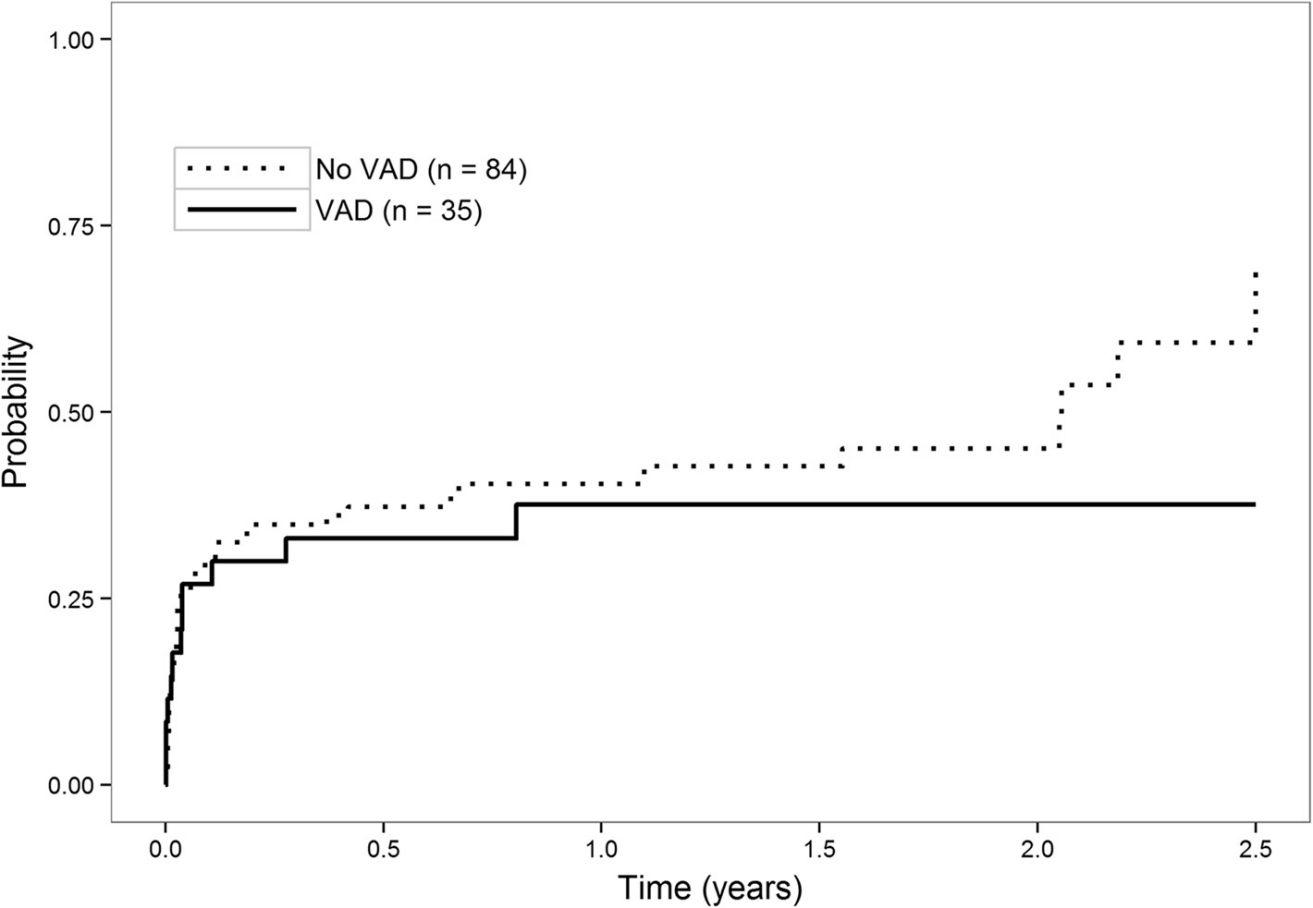
Cumulative incidence of bacterial or candida infection after transplantation according to whether the patient had a VAD or not at the time of transplantation

Cumulative incidences of first episode of bacterial or *Candida* infection at one year were 37.7 % in VAD patients and 40.4 % in non-VAD patients (Fig. 2). The most common sites of post-transplant infections were the respiratory tract in patients without VAD (25.7 % of post-transplant infections) and the surgical site in patients with VAD (38.9 %) (Table 4). Other post-transplant outcomes were similar between patients with or without VAD. After one year of follow-up, patient survival was 82.9 % (29/35) in the VAD group and 82.1 % (69/84) in the non-VAD group.

**Table 4:** Outcomes after transplantation in patients with VAD and without VAD

	No VAD (*n* = 84)	VAD (*n* = 35)
Patients with ≥1 post transplant bacterial/candida infection episode, n (%)	39 (46.4)	12 (34.3)
Number of episodes of bacterial/candida infections per patient; mean (SD)	0.83 (1.31)	0.51 (0.82)
Number of post-transplant bacterial/candida infections episodes	70	18
Sites of bacterial/candida infections; n (%)^a^		
- Respiratory tract - Bacteremia - Urinary tract - Surgical site - Other^b^	18 (25.7) 11 (15.7) 12 (17.1) 17 (24.3) 17 (24.3)	4 (22.2) 5 (27.7) 3 (16.7) 7 (38.9) 2 (11.1)
Microorganisms; n (%)		
- *Escherichia coli* - Coagulase Negative *Staphylococci* - *Staphylococcus aureus* - *Pseudomonas sp.* - *Clostridium difficile* - *Enterobacter sp.* - *Candida sp.* - Polymicrobial - Other	10 (14.3) 6 (8.6) 6 (8.6) 6 (8.6) 5 (7.1) 6 (8.6) 6 (8.6) 5 (7.1) 19 (27.1)	2 (11.1) 1 (5.6) 1 (5.6) 0 2 (11.1) 2 (11.1) 1 (5.6) 3 (16.7) 7 (38.9)
Viral infections		
- CMV infection - VZV infection - HSV infection	31 (36.9) 8 (9.5) 15 (17.9)	8 (22.9) 3 (8.6) 1 (2.9)
Other fungal infections		
- *Aspergillus spp.*infection - *Pneumocystis jiroveci* pneumonia	7 (8.3) 3 (3.6)	1 (2.9) 0 (0.0)
Length of stay after transplant, days; mean (SD)	60.8 (65.0)	82.9 (127.6)
Patients with treated acute rejection, n (%)	57 (67.9)	22 (62.9)
Survival; n (%)	69 (82.1)	29 (82.9)

There were no significant differences between groups

*VAD* ventricular-assist device, *CMV* cytomegalovirus, *VZV* varicella-zoster virus, *HSV* herpes simplex virus

^a^One episode of infection could be counted multiple times according to the number of infectious sites

^b^Other infections: 8 gastro-intestinal infections, 5 muco-cutaneous infections, 4 bone/joint infections, 2 catheter-related infections

In multivariate analysis, cotrimoxazole prophylaxis (HR 0.29, [95 % CI [0.15-0.57], *p* < 0.001) was the only variable associated with bacterial or *Candida* infections, but VAD was not (HR 0.94, [95 % CI 0.38-2.32], *p* = 0.89, for continuous-flow devices, and HR 0.45 [0.15 – 1.34], *p* = 0.15, for other devices) (Table 5). We did not observe any influence of VAD on the incidence of all fungal infections (HR 0.27 [95 % CI 0.06-1.30], *p* = 0.10) or viral infections (HR 0.91, [95 % CI 0.32-2.56], *p* = 0.86, for continuous-flow devices, and HR 0.64 [0.18 – 2.22], *p* = 0.48, for other devices) (Table 5).

**Table 5:** Multivariate analysis of risk factors for infection after heart transplantation

	Bacterial and candida infection			All fungal infections			Viral infections		
	Hazard ratio	95 % CI	*p*-value	Hazard ratio	95 % CI	*p*-value	Hazard ratio	95 % CI	*p*-value
Recipient age^a^	0.89	0.67-1.19	0.43	1.30	0.77-2.21	0.32	0.91	0.68-1.20	0.49
Recipient male gender	2.06	0.85-5.01	0.11	0.98	0.22-4.32	0.98	1.59	0.62-4.10	0.33
Donor age^a^	1.03	0.81 – 1.31	0.81	1.10	0.74 – 1.63	0.64	0.92	0.70 – 1.20	0.54
Donor male gender	1.88	0.91 – 3.89	0.09	0.76	0.27 – 2.20	0.62	0.50	0.23 – 1.06	0.07
VAD pre-transplant				0.27^b^	0.06-1.30	0.10			
- Continuous-flow device	0.94	0.38- 2.32	0.89				0.91	0.32 – 2.56	0.86
- Other device	0.45	0.15 - 1.34	0.15				0.64	0.18 – 2.22	0.48
Diabetes	1.48	0.69-3.20	0.32	2.74	0.87-8.57	0.08	2.01	0.82-5.31	0.12
Post-transplant cotrimoxazole prophylaxis	0.29	0.15-0.57	<0.001	1.04	0.36-3.01	0.95	1.03	0.49-2.17	0.94
Transplant center									
Center A (ref.)	1			1			1		
Center B	0.69	0.32 – 1.49	0.35	1.38	0.40-4.78	0.61	1.06	0.46-2.45	0.89
Center C	0.55	0.23 – 1.31	0.18	0.28	0.04-1.78	0.18	0.50	0.18-1.44	0.20
Induction therapy									
No induction(ref.)	1			1			1		
ATG	1.01	0.35 – 2.96	0.98	0.18	0.02-1.37	0.10	1.20	0.34-4.25	0.78
Basiliximab	1.57	0.42 – 5.92	0.50	0.40	0.03-5.58	0.50	-	-	-
Use of everolimus	0.34	0.08 – 1.49	0.15	2.24	0.54-9.33	0.27	0.90	0.28-2.86	0.86

*VAD* ventricular-assist device, *ATG* anti-thymocyte globulin

^a^For ten years age difference

^b^No patient in the “other device” group developed a fungal infection, so the specific hazard ratio for this subgroup could not be calculated

### Impact of pre-transplant VAD infection on post-transplant outcomes

Additional file 1: Table S1 shows the clinical characteristics of the 18 patients with pre-transplant VAD-specific or related infection. Six of them developed 10 post-transplant infections. Out of these 10 infections, four episodes were surgical site infections, three primary bacteremias and three respiratory tract infections. Additional file 1: Table S1 also shows the 6 reported post-transplant deaths in VAD group. All of them occurred in patients who had a pre-transplant VAD-specific or related infection (patients #2, #4, #10, #14, #17, #18). Therefore, death during the first year post transplant occurred in 6/18 patients (33.3 %) with pre-transplant specific or related VAD infection and in 0/17 (0 %) in patients without pre-transplant VAD infection. Mean age was 59 years (SD 8.57) in patients who died as compared to 51 years (SD 7.08) in those who survived. There were no significant differences in the induction and maintenance immunosuppressive regimen in these two groups. In 3 of these 6 patients the infection was considered to be cured and in 3 patients the infection was considered to be active. Accordingly, 3 out of the 6 patients were receiving antibiotic therapy at the time of transplant against the pathogen responsible for the VAD infection. Five of these 6 patients died within the first week after transplantation. In one of these 6 cases, infection was considered the direct cause of death (patient #3, multiorgan failure). The patient was bacteremic with *Proteus spp* and *Klebsiella spp* that were also found on culture performed from the pus at the VAD site. Effective antibiotics were given at the time of transplant, but the clinical situation was complicated by a colic ischemia and a multi-organ failure followed by death. The other 5 patients died of non-infectious causes (1 hemodynamic failure, 2 graft dysfunctions, and 2 post surgical hemorrhages).

## Discussion

In this nationwide cohort of heart transplant recipients, we assessed the impact of the pre-transplant implantation of VAD on the incidence of post-transplant infections. We were interested in investigating whether patients who underwent transplantation with a VAD had a higher incidence of post-transplant infections, specifically in patients with a VAD infection before transplantation. We found that VAD was not a risk factor for developing bacterial or *Candida* infection (the main endpoint of the study), or other post-transplant infections. Indeed, the main variable associated with a higher incidence of post-transplant *Candida* or bacterial infection was the lack of cotrimoxazole prophylaxis. However, in the group of patients with VAD, we observed that those with a previous VAD-specific or –related infection had impaired post-transplant survival.

Post-transplant mortality in patients with VAD support has been assessed in several studies, including a large registry from the ISHLT with more than 100,000 heart transplant recipients [11]. In this study, 1-year mortality of patients with continuous-flow left VAD at the time of transplantation was 87 %, as compared to 88 % in patients without left VAD with inotropic support and 90 % in patients without left VAD and without inotropic support. Of note, survival was significantly lower in patients with biventricular VAD (81 % at one year). This is in concordance with the results of our study, as we showed that patients with a VAD in place had similar rate of graft and patient survival. However, other studies have concluded that short-term survival is diminished in patients with a pre transplant VAD [27, 28].

Less data are available regarding the incidence of post-transplant infection in patients with a VAD in place at the time of transplantation. Some studies have described a relative immunosuppressive state in patients with VAD, showing a higher incidence of hypogammaglobulinemia [15] and a decrease in the response of lymphocyte stimulation tests [16] after VAD implantation. Thus it can be hypothesized that these patients might potentially develop more opportunistic infections after transplantation. In our analysis, VAD was not associated with a higher incidence of infection, including bacterial, fungal and viral infections. In line with the results of our study, two additional studies revealed that having a VAD did not increase the risk of post-transplant infections [29, 30]. Indeed, in the study of Drakos et al. [30], patients with a VAD had fewer post-transplant infections than the group transplanted without VAD. This could be related to a cautious administration of immunosuppression at the time of transplant in patients with VAD. In that regard, we found a trend towards a lower frequency of administration of lymphocyte-depleting antibodies in patients with VAD, although in our analysis the type of induction therapy was not associated with a higher rate of infectious complications. It seems clear that immunosuppressive regimens need to be individualized in patients with VAD support, avoiding induction therapy with depleting antibodies particularly in those patients with pretransplant VAD infection.

Because a significant proportion of patients with a VAD could have developed a VAD infection before transplantation, there is some concern that this infection would persist after removal of the device, resulting in post-transplant surgical site infections, mediastinitis, bacteremias and sepsis. In a study involving 149 patients with VAD, 69 % of patients with a pre-transplant driveline infection developed significantly more post-transplant surgical site infections and had a longer length of stay than patients without infection, although post transplant survival was not affected by the presence of a pre transplant infection [14]. In our study only one patient developed a surgical site infection after transplantation with the same microorganism that was responsible for a pre-transplant VAD infection, without disseminated infection. However, in the VAD group, all six patients that died after transplant had a pre-transplant VAD infection. Obviously, patients who had early mortality were not anymore at risk for developing post-transplant infections, so that we try to reduce the bias by applying a competing risk analysis to take death into consideration for the incidence of post transplant infection. Although the cause of death in these patients was only attributed to infection in one case, it is plausible that infection might have actually contributed to mortality. This higher mortality in patients with pre-transplant VAD infection is concordant with other recent studies reporting that infection of VAD had deleterious consequences on allograft and patient survival [13, 31]. Despite that, VAD infection remains an indication for heart transplantation, which it is the only curative treatment in this scenario.

We observed a low rate of specific or related VAD infections caused by antibiotic-resistant pathogens. Of note, we observed that a minority of patients had methicillin-resistant *Staphylococcus aureus* (MRSA) infection [32], with no cases of vancomycin-resistant enterococci (VRE) and extended-spectrum beta lactamases (ESBL)-producing *Enterobacteriaceae* infections. This is in keeping with the current epidemiology of infection at our country. Although we did not have detailed data of the antibiotic resistance patterns after transplant for our study, other studies in the transplant population in Switzerland showed that the incidence of infection caused by resistant pathogens was less than 1 % for VRE (2 episodes out of 392 enterococcal events) [33] and 14 % for ESBL-producing *Enterobacteriaceae* [van Delden et al., manuscript in preparation]. So far, no infections due to carbapenemase-producing bacteria have been recorded in SOT recipients in Switzerland.

We acknowledge that the present study has several limitations. First, as compared to other studies based on a registry, our study has a relatively modest sample size, so we might have not been able to correct our analysis for the different confounders, such as the different antimicrobial and immunosuppressive protocols used at each transplant center. Also, a significant percentage of the VAD used in this study were pulsatile-flow devices, which have a significantly higher risk of becoming infected that the continuous-flow devices, so that different outcomes might have been observed if these new devices would have been used (although we did not identify the use of non-continuous-flow devices as a risk for post transplant infection). However, we used a comprehensive database with standardized definitions and were able to describe in detail the epidemiology of infections after heart transplantation.

## Conclusion

In this nationwide cohort of heart transplant recipients, transplantation with VAD at the time of transplant had no influence on the development of post-transplant infections. However, patients with a history of pre-transplant VAD infection showed a significantly higher early post-transplant mortality.

## Abbreviations

INTERMACS, Interagency Registry for Mechanically Assisted Circulatory Support; SOT, solid-organ transplantation; STCS, Swiss Transplant Cohort Study; VAD ventricular assist device

## Additional file

Additional file 1: Table S1. Clinical characteristics of the 18 patients with a pre-transplant VAD-specific or related infection. (DOCX 16 kb)
