# Supplementary material for: Ventricular assist devices as bridge to heart transplantation: impact on post-transplant infections
Source: BMC Infect Dis. 2016 Jul 8;16:321. doi: 10.1186/s12879-016-1658-0 (PMC4938972; doi:10.1186/s12879-016-1658-0)
Supplement: Additional file 1: Table S1. — Clinical characteristics of the 18 patients with a pre-transplant VAD-specific or related infection. (DOCX 16 kb) [file 12879_2016_1658_MOESM1_ESM.docx]

**Additional file 1: Table S1. Clinical characteristics of the 18 patients with a pre-transplant VAD-specific or related infection**

| **Pre-transplant infections** | | | | | | | | | | **Post-transplant infections** | | | | |
| --- | --- | --- | --- | --- | --- | --- | --- | --- | --- | --- | --- | --- | --- | --- |
| **Patient number** | **Type of VAD** | **Age at transplant**  **(years)** | **Center** | **Time from VAD to transplant (days)** | **Time from VAD to first infection (days)** | **Number of episodes of infection** | **Site of infection** | **Microorganism** | **Ongoing antibiotic therapy at the time of transplant** | **Number of episodes of infection** | **Site of infection** | **Microorganism** | **Time from transplant to infection (days)** | **Outcome at the end of follow-up** |
| 1 | Berlin Heart® EXCOR | 49 | A | 758 | 212 | 11 | Driveline, cannulae, and pump infections with BSI | *S. aureus*, *Enterococcus spp., Enterobacter spp., E. coli, Proteus spp, Bacteroides spp.* | Yes | 1 | Surgical site | *E.coli, Enterococcus spp., Proteus spp*, Anaerobes | 163 | Alive |
| 2 | Berlin Heart® EXCOR | 46 | A | 720 | 123 | 5 | Driveline, cannulae, and pump infections with BSIs | *S.aureus* MRSA, *E. coli* | No | 2 | Bacteremia  Bacteremia | *Proteus spp*  *Proteus spp, Klebsiella spp.* | 2  4 | Dead (Day 7)  Multi-organ failure due to infection |
| 3 | Thoratec® | 49 | B | 112 | 98 | 1 | Mediastinitis | *S. aureus* | No | 2 | Pneumonia  Pneumonia | *Streptococcus spp*  Gram negative bacteria | 596  777 | Alive |
| 4 | Berlin Heart® EXCOR | 54 | C | 177 | 20 | 1 | Mediastinitis | No pathogen | No | 2 | Pneumonia with bacteremia  Bacteremia | Anaerobes  *Corynebacteria* | 101  175 | Dead (Day 180)  Hemodynamic failure |
| 5 | Berlin Heart® EXCOR | 41 | C | 298 | 222 | 1 | Driveline infection | CNS | No | 1 | Surgical site | *Candida spp* | 294 | Alive |
| 6 | Heartmate® II | 51 | A | 517 | 17 | 1 | BSI | *S.aureus* MRSA | Yes | 2 | Surgical site  Surgical site | *Enterococcus spp, Proteus spp,* CNS  *Enterobacter spp* | 14  35 | Alive |
| 7 | Heartmate® II | 52 | A | 337 | 10 | 4 | Driveline and pocket infections with BSI | CNS, *Neisseria* spp. | Yes | 0 | No infection |  |  | Alive |
| 8 | Heartmate®II | 55 | A | 124 | 57 | 2 | BSI | Gr. C *Salmonella, Enterococcus spp* | Yes | 0 | No infection |  |  | Alive |
| 9 | Heartmate®II | 37 | A | 36 | 21 | 1 | Mediastinitis | No pathogen | No | 0 | No infection |  |  | Alive |
| 10 | Thoratec | 63 | A | 401 | 63 | 3 | Driveline infection and CVC-associated BSI | *S.aureus* MRSA and VISA, *C.albicans* | Yes | 0 | No infection |  |  | Dead  (Day 1)  Pulmonary hemorrhage |
| 11 | Heartmate®II | 54 | B | 776 | 186 | 1 | Driveline infection | *S.lugdunensis* | Yes | 0 | No infection |  |  | Alive |
| 12 | Heartware | 52 | B | 161 | 65 | 1 | Driveline infection | *S. pyogenes* | Yes | 0 | No infection |  |  | Alive |
| 13 | Heartmate® II | 55 | B | 89 | 3 | 1 | Driveline infection and BSI | *E.coli, Enterococcus spp* | Yes | 0 | No infection |  |  | Alive |
| 14 | Heartmate® II | 71 | B | 671 | 27 | 2 | Driveline infection and BSI | CNS*, Klebsiella spp* | Yes | 0 | No infection |  |  | Dead  (Day 2)  Graft dysfunction |
| 15 | Berlin Heart®  EXCOR | 48 | C | 62 | 45 | 1 | BSI | *Propionibacterium spp* | No | 0 | No infection |  |  | Alive |
| 16 | Heartmate®II | 65 | B | 289 | 104 | 2 | Driveline infection | *S.aureus, Serratia spp* | No | 0 | No infection |  |  | Alive |
| 17 | Heartmate®II | 62 | A | 567 | 19 | 4 | Pocket infection, mediastinitis and BSIs | *Enterobacter spp,* | Yes | 0 | No infection |  |  | Dead  (Day 0)  Hemorrhage |
| 18 | Berlin Heart®  EXCOR | 57 | C | 219 | 45 | 1 | Driveline infection | No pathogen | No | 0 | No infection |  |  | Dead  (Day 1)  Graft dysfunction |

VAD: Ventricular-assist device; BSI: Bloodstream infection; CVC: Central venous catheter; CNS: coagulase-negative *Staphylococci*; *S.aureus: Staphylococcus aureus*; *E.coli: Escherichia coli; S.lugdunensis: Staphylococcus lugdunensis; S.pyogenes: Streptococcus pyogenes; C.albicans: Candida albicans*
